# Water-Immersion Stability of Self-Compacting Potassium Magnesium Phosphate Cement Paste

**DOI:** 10.3390/ma16062183

**Published:** 2023-03-08

**Authors:** Yuying Hou, Lin Li, Tao Li, Qianqian Wu, Yali Zhou, Jianming Yang

**Affiliations:** 1 Department of Civil Engineering, Sanjiang University, Nanjing 210012, China; hou_yuying@sju.edu.cn (Y.H.); yglitaotao@163.com (T.L.); wu_qianqian@sju.edu.cn (Q.W.); 002209@sju.edu.cn (Y.Z.); 2 China Construction Eighth Bureau Third Construction Co., Ltd., Nanjing 210012, China; lilin3195@163.com

**Keywords:** magnesium potassium phosphate cement (MKPC), self-compacting, water corrosion, strength, volumetric deformation

## Abstract

For the repair of narrow cracks in concrete, the potassium magnesium phosphate cement (MKPC)-based material paste should have high fluidity and self-compacting ability, making it convenient for pouring and compacting. A self-compacting MKPC paste that meets the index requirements recommended by the European Federation of National Associations Representing for Concrete (EFNAFC) was prepared by increasing the water–cement ratio and adding water glass and fly ash (FA). Specimens of self-compacting MKPC paste were subjected to long-term water corrosion tests, which found that those high-fluidity MKPC paste specimens (reference sample M0) that were produced with only an increased water–cement ratio lost 15–30% of their strength. The residual ratio of folding to compression was 84.6%, and the volume expansion rate was 7.78 × 10^−4^ after immersion in water for 560 days. The strength residual rate of MKPC slurry (M1) modified by sodium silicate and fly ash is over 90% after 560 days of immersion in water, and the residual rate of flexural-compressive ratio is 101.3%, which meets the requirements of hydraulic cement-based materials. The volume expansion rate of M1 is 5.19 × 10^−4^, which is 67% of the reference sample M0 with the same water immersion age.

## 1. Introduction

Magnesium phosphate cement (MPC) is a type of cement composed of dead-burned magnesia powder, soluble phosphate and additives in certain proportions, which is mixed with water under acidic conditions and undergoes an acid–base neutralization reaction that forms a phosphate gel phase as the main product [1]. Inorganic cementitious material has the advantages of rapid setting, high early strength, strong adhesion and compatibility with substrates (concrete, steel, wood, etc.), good volumetric stability, low permeability and strong salt corrosion resistance [2,3,4,5,6,7,8,9,10]. Research on MPC systems has been carried out for more than 50 years. It has gradually been applied to biological, structural and refractory materials, the curing of toxic substances and solid waste, the rapid repair of roads and bridges, and military engineering [1,2,3,4,5,6,7,8,9,10]. Research on its civil engineering applications has mainly focused on the repair of concrete structures [2,3,6,7] and the prevention of corrosion in reinforced concrete/steel structures [11,12,13,14]. If it is used to repair narrow concrete cracks, MPC-based material paste needs to have high fluidity and a self-compacting ability that makes it convenient for pouring and compacting. Similarly, MPC-based coatings used for spray construction require high fluidity and thixotropy. These applications place higher demands on the rheological properties of MPC pastes. Existing MPC-based repair materials are mainly used for the repair of large, three-dimensional defects in concrete structures. This kind of repair does not require an MPC-based repair material with good workability [2,3,6,7]. MPC paste is acidic and has a low water–cement ratio. Water-reducing agents and plasticizers suitable for Portland cement have no obvious effect on the fluidity of MPC paste. The fluidity of MPC paste is more dependent on the quality and fineness of dead-burned magnesium oxide, the magnesium–phosphorus and water–cement ratios, and the additives used [15,16,17,18]. Based on existing research [15,16,17,18], the researchers involved in the present study improved the fluidity and workability of fresh MKPC paste q by adjusting the acid–base ratio, increasing the water–cement ratio, and adding water glass and fly ash. A self-compacting MKPC paste [19] was prepared that meets the EFNARC design specification for self-compacting concrete [20].

As a repair and coating material, self-compacting MKPC paste is used in wet environments, so its long-term water stability needs to be evaluated. Existing studies on the water stability of MPC systems [21,22,23,24,25,26] have mainly used MPC-based materials with low water-to-cement ratios and short immersion periods (≤ 12 months). The water in an MPC system is an important solvent involved in the hydration reaction of the reactants in fresh paste and is also an important component of hardened paste. Its dosage has a certain impact on the morphology, type and microstructure of hydration products [27,28,29]. A high water–cement ratio can significantly improve the fluidity of fresh MPC paste but has an adverse effect on the microstructure of hardened MPC paste which, in turn, affects its permeability resistance and water stability [27,28,29]. So far, there are few reports on the performance of self-compacting MKPC pastes under long-term water immersion. Accordingly, the present study aimed to configure MKPC pastes with high fluidity and self-compacting functionality and study their long-term performance during water immersion experiments. Their performance variation with time in a corrosive water environment and an evaluation of their water stability provide the basic data required for the application of high-fluidity, self-compacting MKPC pastes used in such environments.

## 2. Materials and Methods

### 2.1. Raw Materials

The MgO content of the dead-burned magnesia powders was ≥92.58%, the average particle size was 60.06 μm, the specific surface area was 216 m^2^·kg^−1^, and the specific gravity (the solid density after removing the gaps between particles of powder material) was 3.11 g/cm^3^. The KH_2_PO_4_ content of food-grade potassium dihydrogen phosphate (KDP) was ≥98%. The composite retarder (CR) used was composed of disodium hydrogen phosphate dodecahydrate, borax and inorganic chloride salt, all of which were analytically pure. The Baume degree of the water glass (WG, liquid sodium silicate) was 39.2 and its modulus was 3.2. The fly ash (FA) used was first-class ash with an average particle size of 45.12 μm.

### 2.2. Preparation of Pastes and Specimens

Based on existing research [19], the dead-burned MgO powder, KDP, CR, WG and FA were weighed according to the mixing ratio in Table 1. To investigate the effect of WG and FA on the water stability of MKPC paste (M1), a reference group M0 was added. Referring to the index requirements of self-compacting mortar recommended by EFNARC (flow expansion = 240–260 mm, V-funnel outflow time = 7–11 s) [20], the flow expansion and V-funnel outflow time of the MKPC paste prepared by increasing the water–cement ratio only within 30 min cannot meet the European Design Specification for Self-Compacting Concrete (EFNARC). After adding an appropriate amount of WG (2%) FA to the MKPC, the fluidity and the viscosity of the fresh MKPC paste were improved within 30 min, and its flow expansion and V-funnel outflow time met the index requirements of the self-compacting mortar recommended by EFNARC. It also exhibited a self-compacting function.

Using a JJ-5 cement mortar mixer to stir the MKPC paste, KDP and CR were first put into the stirring pot. Then, part of the water and WG were poured in and slowly stirred for 1 min. Then, dead-burned MgO powder, FA and the remaining water were added and slowly stirred for 1 min, then rapidly stirred for 3–4 min. The paste was poured into a test mold and a knife used to agitate the paste several times to remove excess air bubbles. Then, the excess paste was scraped from the surface with a spatula. The surface of the molded specimen was covered with plastic wrap to prevent moisture from evaporating, which was removed after 5 h. Both the mold and die-removed specimens were placed in a curing room (temperature = 20 ± 2 °C, relative humidity = 60 ± 5%) for 28 d, then soaked in water until the specified age. Three MKPC test pieces of different sizes were prepared in this experiment, which are respectively 25 mm × 25 mm × 280 mm prisms (for deformation testing), 40 mm × 40 mm × 160 mm prisms (flexural, compressive strength testing) and 100 mm high × 50 mm diameter cylinders (mass testing). The strength development of the MKPC specimens in the curing room is shown in Table 2. 

### 2.3. Test Methods

Referring to EFNARC’s recommended workability test method [20], the ambient temperature was controlled at 20 ± 2 °C. The MKPC paste preparation method was the same as that used for the molded specimen. Fresh MKPC paste (6 min after the addition of alkali components) was poured into a frustoconical round mold all at once, the air bubbles in the paste were removed and the surface smoothed, then the frustoconical round mold was immediately lifted vertically to make the paste flow freely. After the paste stopped flowing on the plane, a steel ruler was used to measure the diameter of the paste in the vertical direction (to obtain the flow expansion, see Table 1①). Similarly, we poured the fresh MKPC paste into the V-shaped funnel at one time. After filling, we removed the air bubbles in the paste and smoothed the surface. We opened the baffle at the mouth of the funnel to allow the paste to flow out freely and used a stopwatch to record all the paste from the funnel and the time for complete outflow (see Table 1①). After letting the MKPC paste stand for 30min (starting from the time when the alkali component was added), it was stirred with a mixer for 2 min, and the flow expansion and outflow time of the MKPC paste from the V-shaped funnel were re-tested (see Table 1②).

Table 2 confirms that the self-compacting MKPC paste specimens (M1) had limited strength development after 28 d. Referring to standard GB/T50082-2009, a long-term water immersion test of the MKPC specimens was started after 28 d of curing in the curing room. During the experiment, in order to keep the pH of the soaking liquid stable, the water was replaced every 30 days. The distance between specimens was 20 mm and the water covered the specimens by at least 10 mm. The specimens were taken out at the set soaking age. A towel was used to absorb the moisture on their surfaces, and then their flexural and compressive strengths, weights and lengths were measured. 

Referring to the Inspection Method of Cement Mortar Strength (GB/T17671-1999), the flexural and compressive strengths of the MKPC specimens were tested by a WED-300 universal testing machine. The loading speed of the flexural strength test was 50 ± 10 N/s, and that of the compressive strength tests was 2400 ± 200 N/s. The formula for calculating the strength loss rate ($\Delta f_{c}$) after immersion in water for different times was as follows:

(1) $$\Delta f_{c}=\frac{f_{c0}-f_{cn}}{f_{c0}}\times 100\%$$

where $f_{c0}$ is the flexural or compressive strength of the MKPC specimen in the saturated state (water immersion for 2 d, MPa) and $f_{cn}$ is the flexural or compressive strength (MPa) of the specimen at an immersion age of *n*. 

Referring to the Cement Sand Dry Shrinkage Test Method (JC/T 603-2004), a large-diameter micrometer was used as the test instrument. The length of the saturated surface dry specimens soaked for 2 d was used as the initial length, and the length was measured at each specified soaking time. The dry shrinkage rate was calculated as:

(2) $$S_{n}=\frac{\left(L_{0}-L_{n}\right)}{250}\times 100\%$$

where $L_{0}$ is the initial length of the specimen (mm), $L_{n}$ is the length of the specimen after *n* days of immersion (mm) and 250 is the effective length of the specimen (mm). 

Taking out the specimen (cylinder) at the specified soaking age, we first absorbed the moisture on the surface of the specimen with a towel, and then weighed the wet mass of the specimen with an electronic balance. We put the MKPC specimen into a closed vacuum drying box, set the oven temperature to 50 °C, turned on the vacuum pump to vacuum after the specimen reached the set temperature, and loosened the suction valve after vacuum drying for 48 h until the normal pressure in the box was restored. We took the MKPC specimen out of the vacuum drying box and weighed the mass of the specimen with a precision electronic balance. We continued the vacuum drying process, repeated the weighing process every 1 h, and calculated the mass change rate of the two adjacent weighing processes. When the relative mass change rate of an MKPC specimen is less than 0.1%, its drying is considered to have reached a constant weight state. The water absorption was calculated according to JGJ/T70-2009 Standard for Basic Performance Test of Construction Mortar, as:

(3) $$\rho_{n}=\frac{m_{n}^{*}-m_{n}}{m_{n}}\times 100\%$$

where $m_{n}^{*}$ and $m_{n}$ are the wet and dry masses of the specimen at *n* days of immersion (g), respectively.

After the strength was measured, specimens of the corresponding soaking age were sampled and soaked in absolute ethanol to terminate the hydration reaction. Before the test, the MKPC samples were first removed from the ethanol, dried to a constant weight, and then processed. The morphology of the hydration products of the MKPC samples was observed by a transmission electron microscope (TEM; jem-2100f) and the selected elemental composition was analyzed by EDS. The phase composition of the MKPC samples was determined by X-ray diffractometry (XRD; Japanese D/max RB X-ray diffraction instrument).

## 3. Results and Discussion

### 3.1. Strength Development 

**Figure 1:**
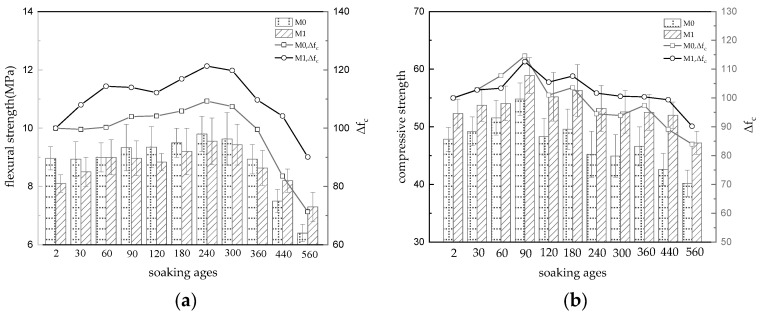

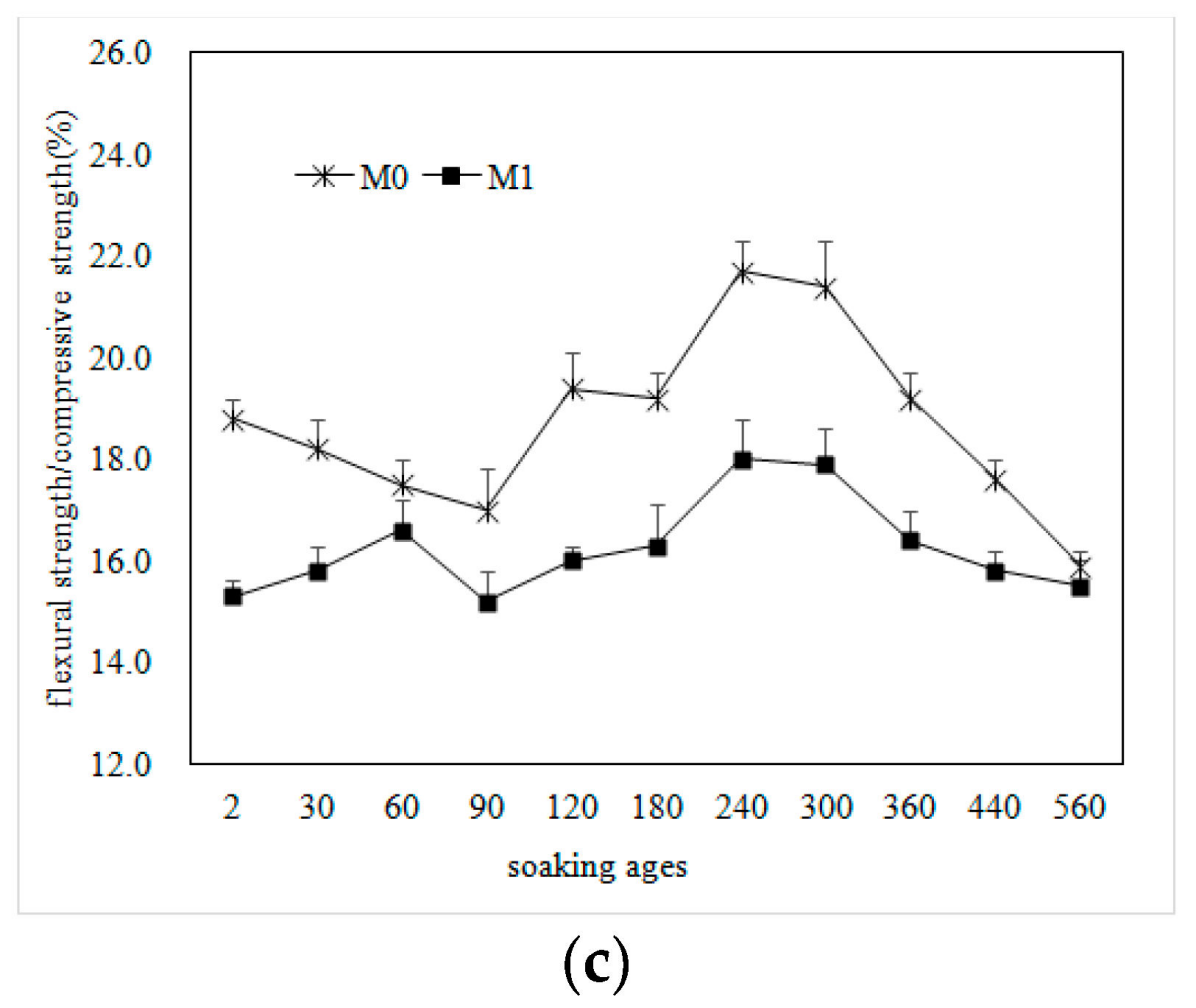
Strength development of MKPC specimens with different water immersion ages. (**a**) flexural strength (**b**) compressive strength (**c**) folding–compression ratio.

Figure 1 shows the strength development of the reference sample M0 and the modified sample M1 during long-term water immersion. The flexural strengths (8.97 MPa and 8.10 MPa) of samples M0 and M1, soaked in water for 2 days after 28 days of natural curing (Figure 1a), were taken as the initial strength (*f_c_*_0_) of the water-saturated specimens. With ongoing water immersion, the flexural strength of the MKPC specimens increased gradually, with the increase in M1 being much greater than that in M0. At an immersion age of 240 d, the flexural strength of the specimens began to decrease, with the decrease in M0 being greater than that in M1. At an immersion age of 560 d, the flexural strength loss rates in M0 and M1 were 71.3% and 90.1%, respectively. According to Figure 1b, the initial compressive strengths of M0 and M1 were 47.83 MPa and 52.30 MPa, respectively. At the initial stage of water immersion, the compressive strength of the MKPC specimens showed an increasing trend, and the increasing range of M1 was larger than that of M0. When the soaking age reached 90d, the compressive strength of M0 and M1 specimens began to decrease, and the decreasing range of M0 was larger than that of M1. When the soaking age reached 560d, the residual rates of compressive strength of M0 and M1 were 84.0% and 90.2% respectively. The results show that the high-fluidity MKPC paste that was obtained by solely increasing the water–cement ratio lost strength during long-term water immersion, while the strength of the paste modified by WG and FA was significantly improved. At an immersion age of 560 d, the residual strength of specimen M1 still exceeded 90%, which meets the requirements of hydraulic cement-based materials. 

The folding–compression ratio can indirectly characterize the crack resistance and cohesion of cement-based materials. Figure 1c shows that the folding–compression ratio of M0 and M1 is 18.8% and 15.3%, respectively, when soaked in water for two days, which is much higher than that of ordinary concrete (1/8~1/12), indicating that it has good crack resistance, but that the addition of fly ash reduces the crack resistance of the MKPC-hardened body. With the prolongation of water immersion age, the folding–compression ratio of M0 and M1 showed a fluctuating growth trend, and the folding–compression ratio reached the maximum when the water immersion age was 240 d. After that the folding ratio of M0 and M1 began to decrease but the decreasing speed of M0 was higher than that of M1. When soaked in water for 560 d, the folding ratio of M0 and M1 was close to (15.9% and 15.5%), and the residual ratio of folding ratio was 84.6% and 101.3% respectively. The results show that adding fly ash can obviously improve the crack resistance stability of an MKPC-hardened body in a long-term water immersion environment.

The water–cement ratio of the self-compacting MKPC paste was 0.16 (Table 1), with the crystal water in borax and the disodium hydrogen phosphate dodecahydrate in the composite retarder component, its actual W/KH_2_PO_4_ value was close to the complete theoretical calculated value upon hydration (0.661 [30]). This shows that the water in the fresh MKPC paste could ensure sufficient hydration of the paste. However, because of the retarding effect of the composite retarder, there were still some unreacted acid–base components in the MKPC specimen in the first 28 days (See TG analysis for details). The strength of the MKPC specimen immersed in water for two days was taken as f_c0_ to eliminate the strength difference between the dry and wet specimens. In the initial stage of immersion, free water penetrated the hardened MKPC paste, which promoted the continuous hydration of the acid and alkali components. The newly generated hydration products filled the pores in the hardened MKPC paste so that the strength of the MKPC specimen continued to increase. The strength of the MKPC specimen soaked in water for 30 days was higher than that of the specimen in the curing room (see Table 2 and Figure 1). With the increase of the amount of cementitious hydration product MKP, the hydration product film adsorbed on the surface of the magnesium oxide particles is thickened and the adhesion is enhanced, which leads to an enhancement of the cohesion of the MKPC-hardened body and an increase of the folding–compression ratio. With consumption of the acid component (KH_2_PO_4_), no new hydration products were generated. However, due to the increasing amount of free water penetrating the hardened body, potassium ions on the surfaces of MKP crystals (composed of H-bonds, K-O, P-O and Mg-O ion covalent bonds) were dissociated from K-O bonds and diffused into the solution. At the same time, the structural water of the MKP crystal lost its chemical stability due to the exchange of weak hydrogen bonds with adjacent free water. With the loss of potassium ions and structural water inside the crystal, the local magnesium ion structure of the crystal and phosphate tetrahedron was distorted, and cracks appeared inside the crystal (dissolution process). After dissolution of the hydration product, the exposed unreacted magnesium oxide continued to be hydrolyzed. Due to the lack of K^+^ and phosphate, the ions in the liquid phase combined to form Mg_3_(PO_4_)_2_·22H_2_O (phase transition) and Mg(OH)_2_ (hydrolysis; See TG and SEM analysis for details). The dissolution and phase transition of MKP crystals and the continuous hydrolysis of unreacted magnesium oxide led to the weakening of the bonding forces between the crystals in the microstructure and between the unreacted magnesium oxide particles, leading to decreased strength and folding–compression ratio in the hardened MKPC paste. Adding WG may increase the actual water-to-binder ratio of the paste and the pH of the liquid phase, thereby improving the fluidity of MKPC paste [18]. The average diameter of FA particles is smaller than that of MgO particles, and most are glass spheres. Adding an appropriate amount of FA into MKPC paste can reduce its viscosity and improve its fluidity. Spherical FA particles themselves have high strength and, as micro-aggregates are filled in the middle of MgO particles, form a good gradation with unhydrated MgO particles, improving the pore structure of the hardened MKPC paste. The cations in FA react with phosphate to form a new gel phase, Ca_3_(PO_4_)_2_, which fill the pores in the hardened paste and make its structure more compact (See XRD analysis for details). However, the proper amount of fly ash replaces magnesium oxide powder, which reduces the effective alkali component of MKPC slurry and the amount of sticky hydration phase MKP generated in the hardened body during hydration for 28 days (See TG analysis for details), in turn leading to a decrease in the cohesion and compression ratio of the hardened body. However, the dense hardened body structure slows down the infiltration rate of environmental water, and the dissolution, phase transformation and hydrolysis of unreacted magnesium oxide in the hardened body of MKPC are alleviated, while the loss of strength and of the ratio of folding to compression is reduced.

### 3.2. Volumetric Deformation 

**Figure 2:**
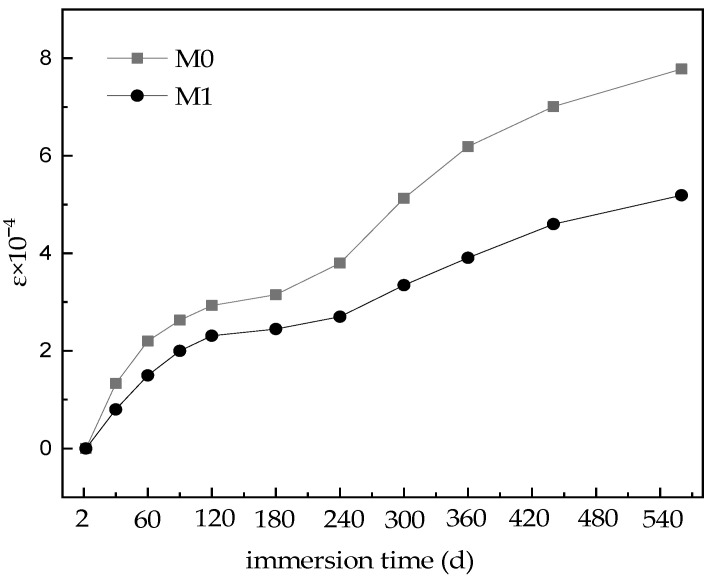
Volumetric deformation of MKPC specimens with water immersion time.

Figure 2 shows the volumetric deformation of the MKPC specimens under long-term water immersion, which indicates continuous expansion. The volumetric expansion rate of the M0 specimens was rapid within the first 60 days, then was stable from 60 to 180 days, and was rapid again beyond 180 days. At 560 days, the volumetric expansion rate was $7.78\times 10^{-4}$. Meanwhile, the volumetric expansion rate of the M1 specimens was rapid within the first 90 days, slower from 90 to 240 days, and increased slightly beyond 240 days. At 560 days soaking age, the volumetric expansion rate was $5.19\times 10^{-4}$, which is only 67% that of the M0 specimens under the same conditions.

**Figure 3:**
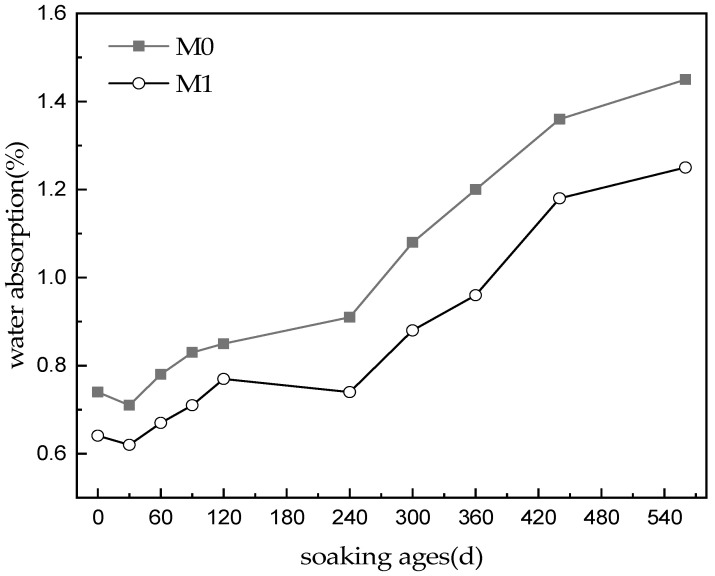
Water absorption of MKPC specimens with immersion time.

**Figure 4:**
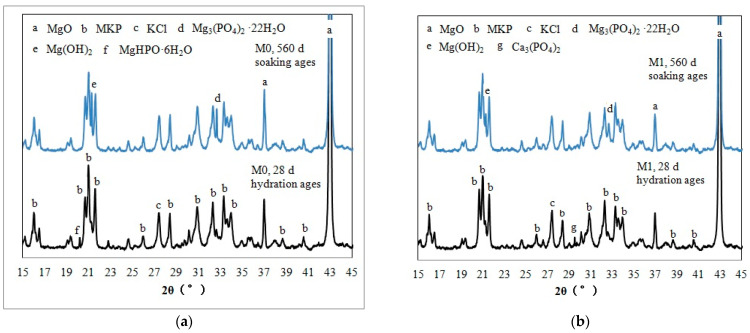
XRD patterns of the MKPC samples. (**a**) M0, (**b**) M1.

At the initial stage of immersion, the volumetric expansion of the specimen M0 was caused by the penetration of free water into the hardened MKPC paste. This promoted the continuous reaction of unreacted acid–base components, with the density of the newly formed hydration product being less than that of dead burned magnesium oxide powder [1,2], resulting in expansion of the MKPC specimens. With completion of the phosphate reaction, the volumetric expansion rate of the MKPC specimen tended to be stable. Afterwards, the hydrated product MKP was dissolved and transformed into Mg_3_(PO_4_)_2_·22H_2_O [30], and the newly exposed magnesium oxide was hydrolyzed into magnesium hydroxide [31]. All of these actions caused the volume of the hardened MKPC paste to expand. Because the structures of the M1 specimens were denser than those of M0 specimens (Figure 3) and the environmental water could not easily penetrate them, the volumetric expansion of the M1 specimens due to continuous hydration in the initial stage of water immersion was significantly less than that of M0 specimens. With further water immersion time, Mg_3_(PO_4_)_2_·22H_2_O was formed by the phase transformation of hydration products and Mg(OH)_2_ was formed by the hydrolysis of magnesium oxide (Figure 4). However, due to the dense structure of M1, which hindered water infiltration, its volumetric expansion rate was significantly lower than that of M0 specimens under the same conditions.

### 3.3. Water Absorption of MKPC Specimens

Figure 3 shows the change in water absorption in the MKPC specimens at different soaking ages. This can be used to characterize the changes in the open pores in hardened paste. The initial water absorption of M0 specimens was 0.74%, which is significantly higher than that (0.53–0.57%) of the hardened paste with a low water–cement ratio (0.113) at the same hydration age [8]. This indicates that increasing the water–cement ratio alone will increase the open pore content of the hardened MKPC paste. Mixing WG and FA reduced the water absorption of hardened MKPC paste (0.64%), indicating that the pore structure of M1 specimens was better than that of M0 specimens. The water absorbed by the two kinds of MKPC specimens decreased slightly when soaked in water for 30 days. After that, the water absorption of the two kinds of MKPC specimens did not change significantly within the first 240 d of immersion. With further immersion, the water absorbed by both kinds of MKPC specimens gradually increased. At 560 d, the water absorbed by M0 was 1.45%, while that of M1 was 1.25%. This indicates that the degree of structural deterioration was lesser in M1 than in M0.

With the infiltration of free water, formation of new MKP, dissolution and phase transformation of existing MKP, and hydrolysis of unreacted magnesium oxide occur in the hardened paste. When the generation and filling of MKP is dominant, the porosity of hardened MKPC paste decreases (e.g., when soaked in water for 30 days). When the formation of MKP and dissolution of existing MKP occur simultaneously and counteract each other, the porosity of hardened MKPC paste remains basically unchanged (e.g., when soaked in water for 30–240 days). When the dissolution of MKP is dominant and is accompanied by the hydrolysis of unreacted magnesium oxide, the internal structure of hardened paste is destroyed, and its porosity increases continuously.

### 3.4. XRD and TG Analyses of Hardened MKPC paste

Figure 4 shows the XRD analysis results of MKPC samples. Figure 4a shows that the main diffraction peaks of the M0 sample, hydrated for 28 d, are MgKPO_4_·6H_2_O(MKP) and unreacted MgO. There is a main characteristic peak of MgHPO_3_·6H_2_O at about 19.9–20.1 2θ°, which should be the hydration product generated in the absence of K^+^. There is a main characteristic peak of KCl at about 27.0–27.6 2θ°, which should be the product formed by the combination of Cl^−^ in the composite retarder and K^+^ in the paste. In the M0 sample immersed for 560 d, characteristic peaks of Mg_3_(PO_4_)_2_·22H_2_O are found at about 32.6 2θ° and characteristic peaks of Mg(OH)_2_ are found at 21.2–21.3 2θ°. This suggests that in the water immersion environment, three ions—Mg^2+^, K^+^ and PO_4_^3−^—dissolve out of MKP, which is the main hydration product in the hardened MKPC paste. When the critical saturation concentration is reached, recrystallization and precipitation occur. Because the solubility of Mg_3_(PO_4_)_2_ 22H_2_O (K_SP_ = 8.0 × 10^−24^) is less than that of MgKPO_4_·6H_2_O (K_SP_ = 2.4 × 10^−11^) [26], it will reach the saturation state first. Similarly, after immersion for a long time, unreacted magnesium oxide particles in the hardened MKPC paste will be exposed by the continuous dissolution of MKP. Under the action of polar water molecules, Mg^2+^ will be hydrolyzed and the pH of the surrounding solution will increase. Mg(OH)_2_ precipitates without gelation will be produced when Mg^2+^ is supersaturated in an alkaline environment. The main diffraction peaks of the M1 sample hydrated for 28 d (Figure 4b) are the hydration products MKP, KCl and unreacted MgO. There is a diffraction peak of Ca_3_(PO_4_)_2_ at about 29.6 2θ°, which should be due to the dissolution and combination of calcium ions in FA with phosphate. In the M1 sample soaked in water for 560 d, there is a characteristic peak of Mg(OH)_2_ at 19.4 2θ°. Mg(OH)_2_ should be formed by hydrolysis of Mg^2+^ from exposed unreacted magnesium oxide and precipitation in an alkaline atmosphere. At about 32.6 2 θ°, there is also a characteristic peak of Mg_3_ (PO_4_)_2_·22H_2_O, which should be formed by recrystallization of Mg^2+^ and PO_4_^3−^ ions dissolved in a water immersion environment.

**Figure 5:**
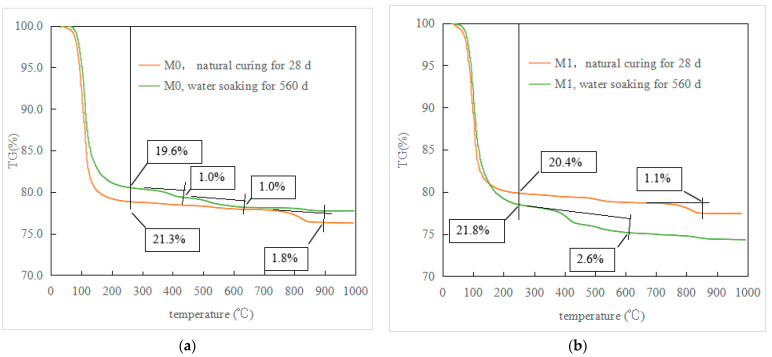
TG curves of the MKPC samples. (**a**) M0, (**b**) M1.

Figure 5a,b show the TG curves of M0 and M1 after 28 d natural curing and 560 d water immersion, respectively. The M0 sample in Figure 5a has obvious mass loss between 60 °C and 200 °C, which is caused by the loss of crystal water from MKP. The weight loss of the M0 sample hydrated for 28 d between 60 °C and 200 °C is 21.3%, which is significantly higher than that of the MKPC sample with a low water–cement ratio [17,18,22,26], indicating that a high ratio is beneficial to MKP generation. The M0 samples at the 28-day hydration age showed a small mass loss between 650 °C and 900 °C, which should be due to the decomposition of hydrogen-containing salts such as MgHPO_4_ [26]. The M0 sample immersed in water for 560 d also has some weight loss between 60 °C and 200 °C (19.6%), which is less than that of the M0 sample before water immersion. This indicates that the dissolution and loss of MKP due to the water immersion process is greater than that of the recrystallized MKP. The M0 sample immersed in water for 560 d has a small weight loss (1.0%) between 300 °C and 450 °C. Compared with the XRD results in Figure 4a, it can be inferred that the decomposition of Mg(OH)_2_ should occur (initial thermal decomposition temperature of Mg(OH)_2_ = 380 °C). There is also a small weight loss between 450–600 °C, which is inferred to be caused by the decomposition of magnesium carbonate. Carbon dioxide in the air can dissolve in ambient water and react with Mg(OH)_2_ to form magnesium carbonate, and the initial decomposition temperature of magnesium carbonate is 540 °C; however, due to its small content, this phase is not present in the XRD results in Figure 4a.

In Figure 5b, the weight loss of the M1 sample hydrated for 28 days between 60 °C and 200 °C was 20.4%, which is slightly less than that of the M0 sample at the same age. The loss of mass between 650 °C and 900 °C should also be due to the decomposition of hydrogen-containing salts. The weight loss (21.8%) of the M1 sample immersed in water for 560 d between 60 °C and 200 °C is greater than that of the M1 sample hydrated for 28 d, indicating that the amount of regenerated MKP is greater than its dissolution and loss. Like M0, the M1 sample soaked in water for 560 d has a small weight loss (2.6%) between 300 and 450 °C, which should be caused by the decomposition of Mg(OH)_2_.

### 3.5. SEM-EDS Analysis of Hardened MKPC Paste

**Figure 6:**
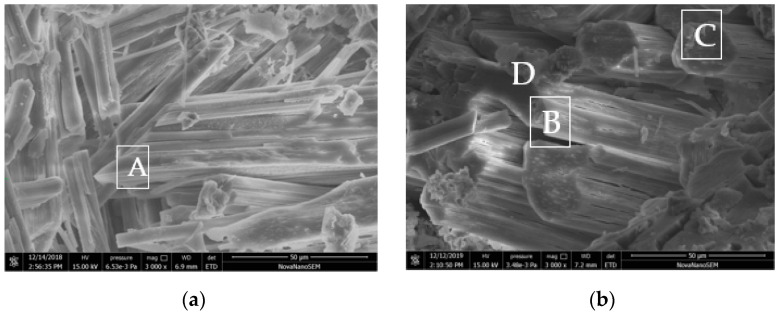

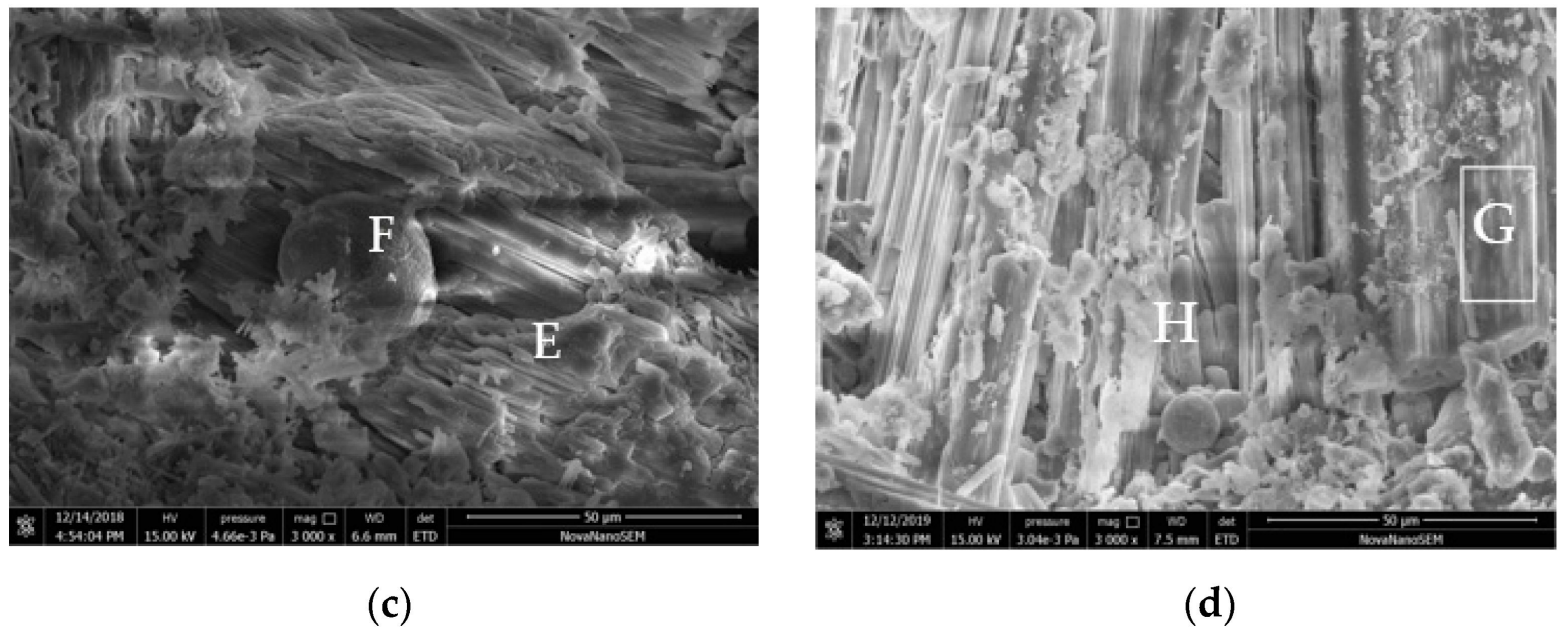
SEM images of MKPC samples. (**a**) M0 natural curing 28 d; (**b**) M0 soaked in water for 36 d; (**c**) M1 natural curing 28 d; (**d**) M1 soaked in water for 360 d.

Figure 6 shows an SEM image of the MKPC sample surface enlarged 3000 times, while Table 3 presents the EDS elemental analysis of the marked area in the image. Figure 6A shows the micromorphology of the hydration products in the cross-sectional hole of the M0 sample after hydration for 28 days. It shows loosely arranged columnar crystals mixed with some needle crystals and many voids between the crystals. The EDS analysis results of micro area A on the surface of the columnar crystal are shown in Table 3. It was composed of Mg, K, P, O, Na and Cl. The molar percentages of Mg, K and P are similar, which should be because MKP (the main hydration product) and Na and Cl came from the CR. Figure 6b shows the micromorphology of the hydration products in the cross-sectional holes of the M0 samples soaked in water for 560 d. The size of the neatly arranged columnar crystals is significantly larger than that of the columnar crystals in Figure 6a, but the crystal surface has obvious corrosion traces, the surface is rough and there are many micro-cracks (see area B), which should be one of the main reasons for the decline in its macro performance. Some small columnar crystals are attached to the surface of the columnar crystals (see area C). The EDS analysis suggests that they are the hydration product MKP, which should be MKP crystals formed by recrystallization. There are some bract-shaped spheres in the gaps between the columnar crystals. EDS analysis of region D confirms that the molar ratio of magnesium and oxygen is close to 1:1.3, while the contents of other phosphorus and potassium plasma are very low (Table 3). Combined with the XRD results, it is inferred that spherical magnesium hydroxide crystals have great expansibility and cause great damage to the structure of the hardened paste. This should be one of the main reasons for the decline of its macro performance.

Figure 6c shows the micromorphology in the cross-sectional holes of the M1 sample hydrated for 28 days. The columnar crystals are neatly arranged, and their size is close to that shown in Figure 6a, but there are many amorphous phases between the crystals and spherical particles fill the gaps between the crystals that have structures significantly denser than those shown in Figure 6a. The EDS analysis of amorphous phase region E are shown in Table 3, which shows the presence of Na, Mg, K, P, O, Si, Al, Ca and Cl. The presence of Al and Ca indicate that cations in the FA were dissolved and participated in the reaction, which is consistent with the XRD analysis results in Figure 4b. Amorphous hydration products are also attached to the surface of the spherical particles. The EDS analysis results of area F are also shown in Table 3, which indicate the presence of Na, Mg, K, O, Si, Al and Ca, which is the basic composition of FA. Figure 6d shows the micromorphology in the cross-section of a hole in the M1 sample soaked in water for 560 days. The hydration products are arranged in a column, but there are obvious signs of corrosion and cracks on the surface of the columnar crystals (see area G), and the corrosion degree is less than that in Figure 6b. Many amorphous phases are attached to the crystal surface and spherical particles are filled between the crystals. The EDS analysis of amorphous phase region H (Table 3) shows a phosphorus–potassium molar ratio close to 1:1, while the content of magnesium is nearly twice as high. This should be the recrystallization product of the hydration reaction caused by the entry of external free water during soaking. The small amounts of Al and Si indicate that these elements from FA are distributed in the hydration products, which also indicates that Al_2_O_3_ participates in the hydration reaction.

## 4. Conclusions

By increasing the water–cement ratio and adding WG and FA, a self-compacting MKPC slurry was prepared that meets the index requirements of self-compacting mortar recommended by EFNARC (flow expansion = 240–260 mm, V-funnel outflow time = 7–11 s). The tests confirmed that strength development was limited after 28 d of hydration. With reference to standard GB/T50082-2009, water corrosion testing of the self-compacting MKPC specimen began after 28 d of hydration. The following conclusions can be drawn from the long-term water immersion test:

The strength loss of MKPC slurry with large fluidity obtained solely by increasing the water–cement ratio reached 15–30% in long-term water immersion environment (560d), and the residual ratio of flexural compression was 84.6%. The high water–cement ratio leads to a large content of open pores in the hardened body, easy infiltration of environmental water, and the dissolution, phase transformation and hydrolysis of unreacted magnesium oxide of MKP crystal, which leads to the weakening of the adhesion between crystals and between crystals and unreacted magnesium oxide particles, and the decrease of the strength and folding–compression ratio of the hardened body. After long-term water immersion, the strength of the MKPC slurry modified by WG and FA was significantly better than the unmodified slurry. After immersion for 560 d, the strength residual rate of the M1 specimen was still > 90%, which meets the requirements for hydraulic cement-based materials. The FA spherical particles that had an average diameter that was smaller than the MgO particles themselves had higher strength and were filled between MgO particles as micro-aggregates. This improved the pore structure of the MKPC-hardened body, while the cations in the FA reacted with phosphate to form a new gel phase Ca_3_(PO_4_)_2_, and AlPO_4_ filled the pores in the hardened body to make the structure more compact. The infiltration rate of ambient water was reduced, there was dissolution of MKP crystals in the hardened body of MKPC, the phase transition and hydrolysis of unreacted magnesium oxide were alleviated, and strength losses were reduced.

The long-term immersion volume stability of self-compacting MKPC paste (M1) modified by WG and FA was significantly improved. After immersion for 560 d, the volumetric expansion rate of M1 was 5.19 × 10^−4,^ which is 67% that of the reference sample M0 (7.78 × 10^−4^) at the same immersion time. The volumetric expansion of the MKPC-hardened body was initially caused by continuous hydration of the incompletely reacted acid and alkali substances, and the density of the new hydration product was less than that of the dead-burned magnesium oxide powder. The second cause was the dissolution and phase transformation of the MKP hydration product and the hydrolysis of the newly exposed magnesium oxide, which formed large, expansive, spherical, magnesium hydroxide crystals. The structure of the self-compacting MKPC paste modified by WG and FA was more compact, the water could not readily penetrate, and the volumetric expansion caused by continuous hydration, phase transition of hydration products and hydrolysis of magnesium oxide was significantly reduced.

## Figures and Tables

**Table 1:** Mix proportions of MKPC paste (wt%).

Code	KDP, CR/ (MgO + FA)	CR/ (MgO + FA)	FA/ MgO	WG/ MKPC	Water/ MKPC	Slump Flow (cm)		V-Funnel Time(s)	
						①	②	①	②
M0	1:2.5	0.125	0	0	0.16	26 × 26	23 × 23	10	25
M1			1:9	0.02	0.16	26 × 26	24 × 24	8	11

**Table 2:** Strength development of MKPC paste specimens in a curing chamber (MPa).

Code	Hydration Age	5 h	1 d	3 d	7 d	28 d	60 d	90 d	180 d
M0	Flexural	3.33	5.67	6.9	6.97	7.86	8.23	8.65	10.1
	Compressive	2.68	28.2	42.2	39.1	44.1	48.3	56.1	60.7
M1	Flexural	3.50	6.57	7.23	7.3	7.9	7.93	8.12	8.56
	Compressive	19.5	33.3	44. 5	41.2	47.3	54.3	55.0	55. 6

**Table 3:** Main elemental contents in regions A–G according to EDS (wt%).

Element	A	C	D	E	F	G
	Mass Ratio					
**Na**	**0.12**		**2.28**	**0.92**	0.50	0.49
Mg	13.76	14.12	37.95	14.04	3.45	9.24
Si				6.30	21.09	10.47
Al				3.55	19.47	5.69
Ca				0.45	0.55	
P	10.01	13.62	2.97	12.90		5.04
K	10.12	14.73	6.15	11.10	1.12	5.85
O	65.90	57.53	49.58	50.74	53.82	62.63
Cl	0.08		1.07			0.59

## Data Availability

No applicable.
